# Evaluation of health behaviors and overall quality of life in younger adult African American cancer survivors

**DOI:** 10.1002/cam4.4855

**Published:** 2022-06-02

**Authors:** Matthew R. Trendowski, Jaclyn M. Kyko, Christine M. Lusk, Julie J. Ruterbusch, Theresa A. Hastert, Felicity W. K. Harper, Hayley Thompson, Jennifer L. Beebe‐Dimmer, Ann G. Schwartz

**Affiliations:** ^1^ Department of Oncology Wayne State University School of Medicine Detroit Michigan USA; ^2^ Karmanos Cancer Institute Detroit Michigan USA

**Keywords:** cancer survivorship, discrimination, health behaviors, health disparities, quality of life

## Abstract

**Background:**

Epidemiological studies of cancer survivors have predominantly focused on non‐Hispanic White, elderly patients, despite the observation that African Americans have higher rates of mortality. Therefore, we characterized cancer survivorship in younger African American survivors using the Detroit Research on Cancer Survivors (ROCS) study to assess health behaviors and quality of life.

**Methods:**

Five hundred and seventeen patients diagnosed with any cancer between the ages of 20–49 (mean age: 42 years; SD: 6.7 years) completed a survey to identify important clinical, behavioral, and sociodemographic characteristics, measures of health literacy, and experiences of discrimination. Quality of life outcomes were evaluated in patients using FACT‐G, FACT‐Cog, and PROMIS® Anxiety and Depression scales. Stepwise linear and logistic regression were used to assess the association between quality of life measures and participant characteristics.

**Results:**

The mean FACT‐G score was 74.1 (SD: 21.3), while the FACT‐Cog was 55.1 (SD: 17.1) (FACT‐G range 0–108 with higher scores indicating better function; elderly cancer patient mean: 82.2; FACT‐Cog 18‐item range 0–72 points with higher scores indicating better perceived cognitive functioning; scores <54 indicating cognitive impairment). In addition, 27.1% and 21.6% of patients had a score indicative of moderate or severe anxiety and depression, respectively. Perceived discrimination and the number of discriminatory events were significantly associated with reductions in three of the four quality of life measures. Health literacy was positively associated with all four health measures, while total comorbidity count was negatively associated with three of the four measures.

**Conclusion:**

Younger adult African American cancer survivors who report experiencing discrimination and suffer from multiple comorbid conditions have poorer mental and overall health. Understanding the unique clinical and socioeconomic stressors that influence this patient population is essential for reducing health disparities and improving long‐term survivorship.

## Graphical abstract

Although there have been improvements in screening and treatment for younger African American cancer survivors that have resulted in decreased mortality, continued differences in mortality between African Americans and Whites are indicative of a health disparities gap. Our study highlights the unique stressors in young‐to‐middle aged adult African American cancer survivors that lead to poor overall and mental health and emphasizes the importance of understanding the social, behavioral, and clinical factors that contribute to the health disparities these individuals face.

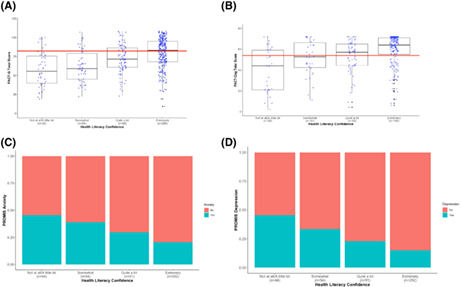

## 1 INTRODUCTION

Cancer is often considered to be a disease with a bimodal distribution, with attention paid predominantly to the pediatric population (<20 years of age) and the older adult/elderly population (50+ years of age). However, the number of patients diagnosed with cancer in early‐to‐middle adulthood (20–49 years of age; termed “younger adults”)^1^ is far greater than pediatric patients, and is one of the leading causes of death per decade for this patient population.^2^ Further, the incidence of several common cancers appears to be increasing in this patient population, including breast, colorectal, and kidney cancer,^3^, ^4^ resulting in more younger adult cancer survivors moving forward. Racial differences in 5‐year relative survival rates are striking, with younger adult African Americans experiencing a 5‐year survival rate of 74.3%, almost 10 percentage points lower than that in Whites (83.5%).^5^

Although there have been previous studies investigating the changes in cancer incidence and mortality among younger adults, evaluation of quality of life for younger adults is lacking particularly among minority and underserved populations. African Americans face a number of adverse effects due to well‐established social determinants of health. Due to higher rates of poverty,^6^ structural racism in healthcare,^7^ lack of access to initial care and patient continuity,^8^ persistent disparities in treatment received in comparison to non‐Hispanic Whites,^9^, ^10^, ^11^, ^12^ and widespread medical mistrust due to numerous historical atrocities,^13^, ^14^ this patient population is uniquely impacted by chronic diseases. Accordingly, it has been repeatedly demonstrated that African Americans have worse treatment outcomes and higher rates of mortality for the most common types of cancer, including when diagnosed as younger adults.^15^, ^16^ Notably, it has been recently demonstrated that African American men younger than 50 have experienced an increase in colorectal cancer incidence, prompting some experts to suggest lowering the initial screening age in this group to 45.^17^ Despite improvements in screening and treatment for this patient population, the disparity in survival between younger adult African Americans and Whites continues.^16^, ^18^

The notable difference in cancer survival between African Americans and other patient populations due to sociodemographic factors and quality of care affect quality of life as patients transition from diagnosis/treatment to survivorship, compromising their long‐term health. Since younger adult African American cancer survivors will likely live years beyond their initial diagnosis, studying a patient population highly susceptible to poorer health outcomes will provide a framework for developing better support mechanisms in this population. The Detroit Research on Cancer Survivors (ROCS) study is a longitudinal study of African American cancer survivors in the Metropolitan Detroit area that examines overall quality of life using physical, social, emotional, and functional measures. In this study, we use the Detroit ROCS dataset to both describe the sociodemographic (age, sex, education, marital status, annual household income, health insurance status, perceived discrimination, health literacy, and religiosity), behavioral (physical activity, smoking status, alcohol consumption, fruit, and vegetable consumption), and health characteristics (cancer site, comorbidities), and evaluate the predictors of quality of life (FACT‐G, FACT‐Cog, PROMIS Anxiety®, and PROMIS Depression®) in younger adult African American cancer survivors.

## 2 METHODS

### 2.1 Study population

The population‐based Detroit ROCS study has assembled a large cohort of African American cancer survivors. The details of this study have been previously described.^19^, ^20^ Briefly, African Americans diagnosed with primary invasive breast, lung, prostate, or colorectal cancer since 2013, and endometrial cancer and any cancer type diagnosed among those ages 20–49 since 2016 while residing in metropolitan Detroit were identified through the population‐based Metropolitan Detroit Cancer Surveillance System (MDCSS) cancer registry. Only individuals diagnosed with breast, prostate, colorectal, and lung cancer ages 20–49 were included during the time frame from 2013 to 2015. To ensure that the sample was representative of all cancer patients in this age range, we limited the analysis to those diagnosed 2016 forward when eligibility included all cancer sites. Survivors were invited to complete the enrollment survey either online, over the phone with a trained interviewer, or by returning a written questionnaire.

As of December 14, 2021, 5008 cancer survivors were enrolled in the Detroit ROCS cohort. The data presented are based on the first 517 younger adult survivors diagnosed from 2016 forward reflecting the start of patient enrollment of the younger cohort across all cancer types. Survivors were approached at least 6 months after diagnosis and as quickly after that time frame as diagnostic data were available in MDCSS. Eligible participants were sent a letter of introduction, consent information, access/password to the online version of the questionnaire, and a paper version of the questionnaire. Trained interviewers contacted eligible participants not completing the survey online or returning the written questionnaire. The survey was completed online (*n* = 144; 27.9%), through the assistance of an interviewer (*n* = 144; 27.9%), or in written form (*n* = 229; 44.3%). Mean time since diagnosis to study enrollment was 19.1 months (SD: 10.6 months).

### 2.2 Study measures

Sociodemographic characteristics at enrollment used in this analysis included age, sex, education, marital status, annual household income, health insurance status, perceived discrimination, health literacy, and religiosity. For sex, males were the reference group for all analyses. Education was defined as: 1 = Less than high school; 2 = High school/GED; 3 = Some college; 4 = 2‐year college degree or higher. Marital status was defined as: 1 = Married; 2 = Living with a partner in a marriage‐like relationship; 3 = Widowed; 4 = Divorced; 5 = Separated; 6 = Never married. Annual household income was defined as: 1 = Less than $20,000; 2 = $20,000–$39,999; 3 = $40,000–$59,999; 4 = $60,000 or more. Health insurance coverage at the time of diagnosis was also self‐reported, and was defined as: 1 = Medicare (with or without supplemental); 2 = Medicaid only, Medicaid+Medicare, or Medicaid+other; 3 = Private only; 4 = Other (VA or other); 5 = No insurance. Insurance status was then categorized as follows for analysis: 0 = Medicare; 1 = Medicaid; 2 = Private only. Other (VA or other) (*n* = 3) and No insurance (*n* = 6) were removed from the analysis due to insufficient sample sizes. To measure perceived discrimination, patients were asked, “Do you feel you have ever personally experienced discrimination?”, with patients responding either 0 = no or 1 = yes.^21^ Health literacy was based answers to the question, “How confident are you filling out medical forms by yourself?” Patients' responses were defined as: 1 = Not at all/A little bit; 2 = Somewhat; 3 = Quite a bit; 4 = Extremely.^22^ Religiosity was evaluated based on the question “How often do you attend church or other religious meetings?” Patients' responses were defined as: 1 = Never/Once a year; 2 = A few times a year; 3 = A few times a month; 4 = Once a week; 5 = More than once a week.^23^

Cancer‐related factors that were obtained from MDCSS included age at diagnosis, diagnosis year, cancer site, and SEER summary stage. Cancer sites presented (Table 1) included those that occurred in at least 5% of participants (otherwise they were grouped as “other”) and hematopoietic cancers were grouped to include lymphoma, myeloma, and leukemia. Treatment status at enrollment was self‐reported.

**TABLE 1:** Clinical and sociodemographic characteristics of younger adult African American cancer survivors (aged 20–49)

	*N*	Column %
Total	517	100.0%
Sex		
Male	125	24.2%
Female	392	75.8%
Age at diagnosis in years		
20–29	38	7.4%
30–39	112	21.7%
40–49	367	71.0%
Mean (SD)	42.0 (6.7)	
Education		
Less than high school	40	7.8%
High school/GED	136	26.6%
Some college	143	27.9%
2‐year degree or higher	193	37.7%
Not reported	5	—
Marital status		
Married	141	27.5%
Living as married	29	5.7%
Widowed	12	2.3%
Divorced	83	16.2%
Separated	19	3.7%
Never married	228	44.5%
Not Reported	5	—
Income		
<$20,000	201	41.8%
$20,000–$39,999	104	21.6%
$40,000–$59,999	65	13.5%
$60,000+	111	23.1%
Not reported	36	—
SEER poverty		
0%– < 5% poverty	27	5.2%
5%– < 10% poverty	61	11.8%
10%– < 20% poverty	109	21.1%
20%–100% poverty	320	61.9%
Not reported	0	—
Insurance status		
Medicare (with or without Supplemental Coverage)	39	7.6%
Medicaid (with or without Medicare/Supplemental Coverage)	233	45.3%
Private Insurance only	233	45.3%
Other	3	0.6%
No Insurance	6	1.2%
Not reported	3	—
Time since diagnosis in months		
Mean (SD)	19.1 (10.6)	
Median (Range)	17.0 (2.0–52.0)	
Cancer site		
Breast	224	43.3%
Cervix uteri	12	2.3%
Colorectal	52	10.1%
Endometrial	21	4.1%
Head and neck	11	2.1%
Hematopoietic^a^	50	9.7%
Kidney	23	4.4%
Lung	15	2.9%
Prostate	39	7.5%
Thyroid	26	5.0%
Other	44	8.5%
SEER summary stage		
Local	257	49.9%
Regional	168	32.6%
Distant	90	17.5%
Unknown	2	—
Cancer treatment status at enrollment		
In treatment	149	29.7%
Finished treatment or no treatment	352	70.3%
Not reported	16	—
Comorbidity count		
0	183	35.6%
1	150	29.2%
2	89	17.3%
3	44	8.6%
4+	48	9.3%
Not reported	3	22.5%
Mean (SD)	1.3 (1.4)	
Obesity		
Yes (BMI ≥ 30)	296	57.9%
No (BMI < 30)	215	42.1%
Not reported	6	—
FACT‐G		
Mean (SD)	74.1 (21.3)	
FACT‐Cog		
Mean (SD)	55.5 (17.1)	
PROMIS ‐ anxiety		
None to slight (<55)	244	50.4%
Mild (55–55.9)	109	22.5%
Moderate (60–69.9)	101	20.9%
Severe (≥70)	30	6.2%
Not reported	33	—
Mean (SD)	53.6 (10.8)	
PROMIS ‐ depression		
None to slight (<55)	308	62.7%
Mild (55–55.9)	77	15.7%
Moderate (60–69.9)	88	17.9%
Severe (≥70)	18	3.7%
Not reported	26	—
Mean (SD)	51.3 (10.1)	

^a^ Hematopoietic includes lymphoma, myeloma, and leukemia.

Participants were also asked if a doctor ever told them that they had any of the following medical conditions: myocardial infarction, congestive heart failure, coronary artery disease, atrial fibrillation, COPD/emphysema, stroke, hypertension, high cholesterol, hepatitis, arthritis, diabetes, or thyroid problems. Participants were considered to have a comorbid condition if they answered in the affirmative to the specific condition. Obesity was defined as a body mass index (BMI; body weight in kg/height in meters squared) of 30 or more calculated from self‐reported height and weight at ROCS enrollment. Health behaviors at enrollment examined in this study included self‐reported smoking, participating in 150 min or more of moderate‐to‐vigorous physical activity per week in the past 4 weeks,^24^ consuming five or more servings of fruits and vegetables per day in the past 4 weeks,^25^ and alcohol consumption in the past 4 weeks. Smoking status was defined as: 0 = current smoker at enrollment; 1 = former smoker at enrollment; 2 = never smoker. Exercise was based on the following two questions: “In the past 4 weeks, how many times each week did you do moderate or vigorous activities on average?”; When you did moderate or vigorous activities in the past 4 weeks, how many minutes did you do each time on average?” Duration of exercise was defined as: 0 = No moderate or vigorous exercise; 1 = Less than 10 min; 2 = 10–19 min; 3 = 20–29 min; 4 = 30–44 min; 5 = 45–59 min; 6 = 60 min or more. Engaging in ≥150 min of physical activity per week was determined by multiplying the number of times exercising per week by the average length of exercise to provide the total average minutes of physical activity per week. This variable was then categorized as follows; 0 = none; 1 = less than 150 min; 2 = 150 min or more. Fruit and vegetable consumption was based on the following two questions: “In the past 4 weeks, how many servings of fruit did you eat per day (not including juices)?”; “In the past 4 weeks, how many servings of vegetables did you eat per day (not including fried potatoes)?” The count of fruit and vegetables consumed per day were then added to find total consumption. The variable was then converted into a binary format: 0 = Less than five servings per day; 1 = 5 or more servings per day. Alcohol consumption was based on the question, “In the past month, have you consumed alcoholic beverages such as beer, wine, or liquor?”, with patients responding either 0 = yes or 1 = no.

Health‐related quality of life (HRQOL) was measured using the Functional Assessment of Cancer Therapy ‐ General (FACT‐G). This measure includes an overall score as well as four subscales (physical, social/family, emotional, and functional well‐being).^26^ The FACT‐G total score is computed as the sum of the four subscale scores, and has a range of 0–108 points. The reliability and validity of the FACT‐G have been extensively documented and used in diverse populations.^27^, ^28^, ^29^, ^30^ Higher scores are reflective of higher quality of life and a 5‐point difference on the total FACT‐G score is associated with a clinically meaningful difference in HRQOL.^31^ Cognitive difficulties in cancer survivors were measured using the Functional Assessment of Cancer Therapy ‐ Cognition (FACT‐Cog). The FACT‐Cog perceived cognitive impairment (PCI) total score can range from 0 to 72 points when using the 18‐item version, with a higher score indicative of better perceived cognitive functioning. For version 3 of the FACT‐Cog, the PCI score has been established as the preferred outcome, with scores <54 indicative of cognitive impairment.^32^

Anxiety and depression were self‐reported using the Patient Reported Outcomes Measurement Information System (PROMIS®) scale (version 4a) that has been validated among the general population, as well as among adults diagnosed with health issues including cancer.^33^, ^34^, ^35^ Scores are normalized to the general population with a mean of 50 and a standard deviation of 10, and higher scores indicate greater anxiety/depression. PROMIS® Anxiety and Depression scores were categorized for severity: none to slight (<55), mild (55–55.9), moderate (60–69.9), and severe (≥70). Scores were dichotomized as: 0 = none to slight/mild or 1 = moderate/severe for both PROMIS® Anxiety or Depression scores to capture clinically meaningful effects.

### 2.3 Statistical analysis

The distribution of survivor characteristics as well as health behaviors and quality of life measures was summarized using counts and percentages for categorical data and means with standard deviations for continuous data. A stepwise regression approach was used to investigate associations between sociodemographic, behavioral, and clinical characteristics and each quality of life measure. Age at diagnosis, sex, and cancer site were selected a priori as covariates due to their previous association with quality of life in cancer survivors.^36^, ^37^ For each outcome, variables significant at *α* = 0.05 in univariate analysis were entered into the stepwise analysis, while the final model included variables significant at *α* = 0.10. Linear regression was used to model FACT‐G and FACT‐Cog scores, while logistic regression was used to model the PROMIS® Anxiety and Depression scores. For all analyses including sex, males were used as the reference group. Analyses were performed in R 3.3.2^38^ and SAS (Cary, NC) version 9.4, with statistical significance set at *p* < 0.05.

## 3 RESULTS

### 3.1 Cohort characteristics

This study provides important descriptive data about sociodemographic (Table 1), behavioral (Table 2), and clinical characteristics (Table 1) for younger adult African American cancer survivors. Of the 517 patients included in the cohort, 125 were male (24.2%), while 392 were female (75.8%). Mean age at diagnosis for all patients was 42 years (SD: 6.7 years). The most common primary cancer diagnosis was breast cancer (*n* = 224; 43.3%). Just under 42% of these survivors reported household incomes of less than $20,000, 61.9% resided in census tracts at 20–100% of the poverty level, and 45.3% received health insurance through Medicaid.

**TABLE 2:** Health behaviors of younger adult African American cancer survivors (Aged 20–49) at enrollment

	*N*	Column %
Total	517	100.0%
Moderate‐to‐vigorous physical activity		
Any physical activity	290	56.3%
No physical activity	225	43.7%
Not reported	2	—
Minutes of moderate‐to‐vigorous physical activity		
None	225	44.5%
<150 min	137	27.1%
≥150 min	144	28.5%
Not reported	11	—
Servings of fruits and vegetables		
Less than 5 per day	361	70.4%
5 or more per day	152	29.6%
Not reported	4	—
Frequency of eating processed mean and other red meat		
Never or less than once per week	101	19.7%
1–3 times per week	115	22.4%
4 or more times per week	297	57.9%
Not reported	4	—
Alcohol use		
Yes	263	51.2%
No	251	48.8%
Not reported	3	—
Smoking status at diagnosis		
Never	352	68.9%
Former	49	9.6%
Current	110	21.5%
Not reported	6	—
Mean pack‐years (SD)	10.0 (8.1)	
Smoking status at enrollment		
Never	352	68.9%
Former	93	18.2%
Current	66	12.9%
Not reported	6	—
Mean pack‐years (SD)	10.4 (8.6)	
Ever personally experienced discrimination		
Yes	265	52.4%
No	241	47.6%
Not reported	11	—
Health literacy: Confidence in filling out medical forms		
Extremely confident	305	60.2%
Quite a bit	95	18.7%
Somewhat	58	11.4%
A little bit	23	4.5%
Not at all	26	5.1%
Not reported	10	—
Religiosity: Frequency of attending church or other religious meetings		
Never	92	20.7%
Once per year	30	6.8%
A few times per year	99	22.3%
A few times per month	61	13.7%
Once per week	110	24.8%
More than once per week	52	11.7%
Not reported/not applicable	73	—

More than half of the cohort (57.9%) met the threshold of obesity (BMI ≥ 30), and while 56.3% reported participating in moderate to vigorous physical activity, only 28.5% indicated that they engaged in ≥150 min of moderate to vigorous physical activity per week. More than 70% of survivors also reported consuming <5 servings of fruits and vegetables per day, and 57.9% reported consuming processed/other red meat 4 or more times per week. A slight majority consumed alcoholic beverages during the past 4 weeks (51.2%). While 21.5% of participants indicated that they were smoking at the time of their diagnosis, only 12.9% were smoking at the time of enrollment. When examining other medical conditions, 331 patients (64.4%) reported having comorbidities, with 181 (35.2%) having multiple comorbidities.

The majority of patients indicated they were either quite a bit or extremely confident that they could complete medical forms by themselves (*n* = 400; 78.9%). In addition, 162 (36.5%) attended church or another religious service at least once per week. Importantly, more than half (265 individuals, 52.4%) of this cohort indicated that they have personally experienced discrimination.

For overall health‐related quality of life, the mean FACT‐G score was 74.1 (SD: 21.3), while the FACT‐Cog was 55.5 (SD: 17.1). In addition, 131 (27.1%) patients had a PROMIS® Anxiety score indicative of moderate or severe anxiety, while 106 (21.6%) had a PROMIS® Depression score indicative of moderate or severe depression.

### 3.2 Associations between sociodemographic factors, clinical characteristics, and health behaviors with quality of life

Using linear and logistic regression, we identified multiple sociodemographic and clinical characteristics associated with quality of life measures (*p* < 0.05) in univariate analysis that were subsequently selected for inclusion in the multivariable modeling (Tables S1 and S2). We performed a stepwise multivariate regression analysis for each quality of life measure, with variables significant at *p* < 0.10 remaining in the final models. Income level (*β* = 4.16, 95% CI: 2.53–5.80, *p* < 0.0001), involvement in religious activities (*β* = 2.15, 95% CI: 0.83–3.47, *p* = 0.002), health literacy confidence (*β* = 5.75, 95% CI: 3.73–7.78, *p* < 0.0001), and engaging in ≥150 min of physical activity per week (*β* = 6.08, 95% CI: 1.79–10.38, *p* = 0.006) were positively correlated with FACT‐G score. A one level increase in income level, a one‐point increase in health literacy confidence, or engaging in physical activity for ≥150 min were each associated with a mean 4–6‐point increase in FACT‐G score. Total comorbidity count (*β* = −3.22, 95% CI: −4.78, −1.66, *p* < 0.0001), and perceived discrimination (*β* = −6.92, 95% CI: −10.60, −3.24, *p* = 0.0002) were negatively associated with FACT‐G score. An increase of one additional comorbidity was associated with a more than 3‐point decrease in mean FACT‐G score, while experiencing any perceived discrimination was associated with an almost seven‐point decrease in mean score (Table 3).

**TABLE 3:** Association of sociodemographic, behavioral, and clinical characteristics with FACT‐G or FACT‐cog scores in multivariate analysis

	β (95% CI)^a^	*p*
FACT‐G		
Income level	4.16 (2.53, 5.80)	<0.0001
Involvement in religious activities	2.15 (0.83, 3.47)	0.002
Health literacy confidence (one point increase)	5.75 (3.73, 7.78)	<0.0001
Engaged in ≥150 min of physical activity per week		
None	Ref	Ref
<150 min	1.68 (−2.89, 6.26)	0.47
≥150 min	6.08 (1.79, 10.38)	0.006
Total comorbidity count	−3.22 (−4.78, −1.66)	<0.0001
Ever perceived discrimination	−6.92 (−10.60, −3.24)	0.0002
FACT‐Cog		
Involvement in religious activities	1.25 (−0.09, 2.59)	0.07
Health literacy confidence (one‐point increase)	6.77 (4.78, 8.75)	<0.0001
Total comorbidity count	−1.95 (−3.48, −0.42)	0.01
Ever perceived discrimination	−3.67 (−7.44, 0.10)	0.06

a Covariates in the multivariate model included age at diagnosis, sex, and cancer site.

Health literacy confidence (*β* = 6.77, 95% CI: 4.78–8.75, *p* < 0.0001) was positively associated with cognitive functioning, with a one‐point increase in health literacy confidence associated with a nearly seven‐point increase in mean FACT‐Cog score. Involvement in religious activities was also associated with a higher FACT‐Cog score (*β* = 1.25, 95% CI: −0.09‐2.59, *p* = 0.07). Total comorbidity count (*β* = −1.95, 95% CI: −3.48, −0.42, *p* = 0.01) and perceived discrimination (*β* = −3.67, 95% CI: −7.44, 0.10, *p* = 0.06) were negatively associated with cognitive functioning. One additional comorbidity was associated with a two‐point decrease in mean FACT‐Cog, while experiencing discrimination was associated with a greater than three‐point decrease in FACT‐Cog score (Table 3).

Patients who were never married (OR = 1.84, 95% CI: 1.04–3.34, *p* = 0.04) were more likely to have a higher PROMIS® Anxiety score than those currently married (Table 4). Health literacy confidence (OR = 0.65, 95% CI: 0.52, 0.80, *p* < 0.0001), engaging in ≥150 min of exercise per week (OR = 0.54, 95% CI: 0.31–0.92, *p* = 0.03), and perceived discrimination (OR = 1.93, 95% CI: 1.24–3.05, *p* = 0.004) were independently associated with PROMIS® Anxiety scores. Experiencing discrimination was associated with an almost twofold increase in odds of moderate/severe anxiety, while engaging in at least 150 minutes of physical activity was associated with a nearly 50% decrease in the odds of moderate/severe anxiety.

**TABLE 4:** Association of sociodemographic, behavioral, and clinical characteristics with PROMIS® anxiety or depression scores in multivariate analysis

	OR (95% CI)^a^	*p*
PROMIS® anxiety		
Marital status		
Married	Ref	Ref
Living with partner	1.85 (0.66, 4.91)	0.23
Widowed	3.34 (0.83, 2.64)	0.08
Divorced	1.63 (0.78, 3.41)	0.19
Separated	2.45 (0.79, 7.26)	0.11
Never married	1.84 (1.04, 3.34)	0.04
Health literacy confidence (one‐point increase)	0.65 (0.52, 0.80)	< 0.0001
Engaged in ≥150 min of physical activity per week		
None	Ref	Ref
<150 min	1.06 (0.63, 1.77)	0.83
≥150 min	0.54 (0.31, 0.92)	0.03
Smoking status		
Current	Ref	Ref
Former	0.83 (0.39, 1.79)	0.65
Never	0.58 (0.31, 1.12)	0.10
Ever perceived discrimination	1.93 (1.24, 3.05)	0.004
PROMIS® depression		
Income	0.77 (0.61, 0.97)	0.02
Health literacy confidence (one‐point increase)	0.67 (0.53, 0.84)	0.0006
Total comorbidity count	1.34 (1.11, 1.63)	0.003
Ever perceived discrimination	1.78 (1.09, 2.93)	0.02

a Covariates in the multivariate model included age at diagnosis, sex, and cancer site.

Income level (OR = 0.77, 95% CI: 0.61–0.97, *p* = 0.02), health literacy confidence (OR = 0.67, 95% CI: 0.53–0.84, *p* = 0.0006), total comorbidity count (OR = 1.34, 95% CI: 1.11–1.63, *p* = 0.003), and perceived discrimination (OR = 1.78, 95% CI: 1.09–2.93, *p* = 0.02) (Table 4) were associated with the dichotomized PROMIS® Depression score. One additional comorbidity was associated with a 30% increase in the likelihood of moderate/severe depression, while experiencing discrimination was associated with an almost 80% increase in the odds of moderate/severe depression. Conversely, an increase in income level was associated with a 23% decrease in the odds of moderate/severe depression while a one‐point increase in health literacy confidence was associated with a similar decrease (30%) in the odds of moderate/severe depression as measured by PROMIS.

## 4 DISCUSSION

In this study, we were able to characterize sociodemographic, behavioral, and clinical factors for a cohort of younger adult African American cancer survivors, a patient population that has been poorly studied. This patient population has financial and social stressors that increase susceptibility to poorer health outcomes following completion of treatment. Notably, patients who experienced discrimination were more likely to have worse mental and overall health as identified by several FACT and PROMIS scales. Health literacy confidence was highly correlated with better health outcomes for every quality of life measure examined demonstrating the importance of patient education in improving overall health. Engaging in regular physical activity (≥150 min per week) was also associated with better overall health and lower rates of anxiety.

Younger adult African American cancer survivors in metropolitan Detroit have unique financial struggles with 41.8% earning an annual income <$20,000, which is considerably less than the median U.S. salary of $34,248. The Carolina Breast Cancer study has indicated that in models adjusted for age, stage at diagnosis, and treatment received, Black women were more likely to report adverse financial impact attributable to cancer when compared to White women, including income loss, healthcare‐related financial barriers, healthcare‐related transportation barriers, job loss, and loss of health insurance.^39^ Similar findings were identified in a study of the Detroit ROCS pilot dataset in which African American cancer survivors were much more likely to experience financial hardship, limiting care, and cancer‐related debt compared to non‐Hispanic White survivors.^40^

### 4.1 Health behaviors

In our cohort of younger African American cancer survivors, while 56.3% reported participating in moderate‐to‐physical exercise, only 28.5% indicated that they engaged in ≥150 min of moderate‐to‐vigorous physical activity per week, thereby satisfying the weekly standard set by the CDC. This is only marginally better than the 24% of the 1500 cancer survivors (median age at diagnosis: 58.2) reporting ≥150 min per week reported in Detroit ROCS participants before the inclusion of the younger onset cohort.^20^ The lower reported physical activity is in contrast to a study of 1670 survivors from the National Cancer Survivor Transition Study indicating that 33.4% of cancer patients (median age: 62.2) transitioning from active treatment to survivorship exceeded guidelines by 2 or more times.^41^ However, in a recent study of majority non‐Hispanic Whites by Arem et al.,^42^ only 14% of those with a cancer history met physical activity guidelines for aerobic and strength‐based exercise (compared with 21% of those without a cancer history). In the same study, 22% of cancer survivors between the ages of 18–39 met these guidelines, a somewhat comparable, but still lower, percentage as than reported in our cohort of younger adult African American cancer survivors.

The majority of patients consumed <5 servings of fruits and vegetables per day (70.4%), and 57.9% consumed processed/other red meat 4 or more times per week. It is important to note that the fruit and vegetables consumption by this patient population, while still lower than recommended, is above the national average, as only 10% of adults in the United States consume at least 1½ to 2 cups per day of fruit and 2 to 3 cups per day of vegetables.^43^ Consumption of processed meats in this cohort, however, is higher than that in the general population. A recent study of 1137 adults indicated that red meat and poultry is often consumed two to four times weekly (42 and 37%, respectively), while processed meat is consumed once weekly by 30% of adults.^44^ A lack of access to nutritious food is a prominent health issue in the metropolitan Detroit area, as 30,000 people do not have access to a full‐line grocer, while 48% of households are food insecure.^45^ Proper nutrition is particularly important in cancer patients because obesity has been associated with an increased risk of cancer recurrence and mortality.^46^

The percentage of individuals in our cohort who reported smoking (12.9%) or consuming alcohol (51.2%) at enrollment was not greater than the US general population (currently smoking: 14%; consumed alcohol within the last month 54.9%).^47^ By contrast, in a recent study of majority non‐Hispanic Whites between the ages of 18–39, 29% with a cancer history reported current smoking compared with 18% of those without a cancer history.^42^ It was encouraging to note that while 21.5% of participants were current smokers at diagnosis, only 12.9% were still smoking at enrollment suggesting that younger African American cancer survivors are changing behaviors after diagnosis.

### 4.2 Health status

Almost 65% of participants reported one or more comorbidities, and 57.9% had a BMI indicative of obesity, which is markedly higher than the US general population (42.4%) or non‐Hispanic Black adults (49.6%).^48^ When assessing overall health, younger adult African American cancer survivors had a mean FACT‐G score of 74.1 and a mean FACT‐Cog score of 55.5. By contrast, the mean FACT‐G score for elderly cancer patients is 82.2,^49^ and FACT‐Cog scores of <54 were reported in a population that was approximately 80% non‐Hispanic White as indicative of cognitive impairment following cancer treatment,^32^ suggesting that younger African American cancer survivors have poorer overall quality of life than older cancer patients in the general population. Mental health was also a notable concern for these patients as 27.1% patients had a PROMIS® Anxiety score indicative of moderate or severe anxiety (mean score = 53.6), while 21.6% had a PROMIS® Depression score indicative of moderate or severe depression (mean score of 51.3). The mean scores in this cohort are in line with a recent study of 310 cancer patients (median age group: 50–54) in which the mean PROMIS® Anxiety and Depression scores were 53.2 and 50.3, respectively,^33^ providing additional evidence that younger adult African American cancer survivors face similar physical and mental challenges following a cancer diagnosis as older cancer survivors. Financial stress may also be a potential contributor, as we identified income to be associated with PROMIS® Anxiety and Depression scores, as well as FACT‐G score.

### 4.3 Predictors of quality of life

While a number of the health behaviors, such as obesity, have been associated with overall survival, this study focused on the identification of predictors of HRQOL. Across all four domains evaluated, common themes were observed. Overall quality of life, cognitive functioning, anxiety, and depression were all impacted by health literacy and perceived discrimination. Three of the four measures (overall quality of life, cognitive functioning, and depression) were associated with comorbidities. Religiosity was associated with improved overall quality of life and cognitive functioning, while physical activity improved overall quality of life and reduced anxiety.

Perceived discrimination likely has a significant impact on mental health and overall quality of life, as it was associated with all four quality of life measures. Notably, FACT‐G scores were 6.92 (95% CI: −10.60, −3.24) points lower among survivors who reported ever experiencing discrimination, indicative of a clinically meaningful difference. Further, an increased number of discriminatory events experienced by younger adult African American cancer survivors correlated with a worse score for these quality of life measures. In our cohort, 52.4% of individuals indicated that they have personally experienced discrimination. The disparity in cancer care between African Americans and other patient populations is well‐documented, as it has been demonstrated for multiple cancers that African American patients are less likely to receive timely surgical resections and adjuvant therapy for earlier stage diseases, as well as chemotherapy and radiotherapy for advanced disease.^50^, ^51^, ^52^ Importantly, perceived discrimination within healthcare and other settings has been shown to be highly associated with mental health, even after adjusting for sociodemographic and psychosocial variables.^53^ In addition, African Americans who persistently perceive discrimination are more likely to have elevated depressive symptoms in comparison to those who do not perceive longitudinal discrimination.^54^ Therefore, it is plausible that the level of discrimination perceived by younger adult African American cancer survivors may significantly affect their mental health, irrespective of their diagnosis.

Health literacy also appeared to be a major predictor of overall quality of life, as patients who were confident with their health literacy had better scores on all four quality of life measures. It has previously been noted that patients who are more informed about their health are much more likely to engage in healthier lifestyle choices, which subsequently improves their quality of life. Notably, Jayasinghe et al.^55^ demonstrated that low health literacy patients were more likely to engage in unhealthy behavior (smoke, less physical activity, and be overweight), which corresponded to lower physical and mental health with large clinically meaningful effect sizes. Similarly, a recent study of breast cancer survivors reported that health literacy was a notable predictor of physical health‐related and mental health‐related quality of life.^56^ Ensuring that patients are adequately informed of their health status not only enables these individuals to act as their own advocates when making healthcare decisions, but also increases the likelihood of developing healthy lifestyle habits after successful cancer treatment. Cancer survivors who do adopt a healthy lifestyle have better long‐term survival outcomes, as indicated by a lower risk of recurrence^57^ and death.^58^

Finally, involvement in religious activities was positively correlated with FACT‐G and FACT‐Cog score in our cohort of younger adult African American cancer survivors. It has recently been reported that religion/spirituality was positively associated with overall physical health in a meta‐analysis of 101 unique samples encompassing over 32,000 adult cancer patients, indicating that attending to patients' religious and spiritual needs could be a beneficial component of comprehensive cancer care.^59^

Major strengths of our study include the size of our cohort, which allowed us to examine the overall health behaviors and quality of life of younger adult African American cancer survivors, a patient population in which there is a dearth of information. We quantified the overall and mental health of patients using the well‐established FACT and PROMIS® scales and identified predictors of poorer outcomes in this population. This study is also strengthened by being population‐based, and while participants were exclusively from the metropolitan Detroit area, the findings are likely to be generalizable to younger adult African American cancer survivors elsewhere. The 2016 U.S. Census estimates that 84% of African Americans live in urban areas, and therefore, we expect the findings from this Detroit‐based study should be applicable to younger adult African Americans living in other urban areas in the United States.

This study highlights the unique stressors in young to middle‐aged adult African American cancer survivors that can influence health behaviors and overall quality of life. In view of our results, healthcare providers could improve management of these patients by emphasizing the importance of health literacy, as well as diet and exercise in improving the likelihood of long‐term survival while reducing the possibility of recurrence. Acknowledging perceived discrimination in the healthcare setting must also be a priority. Future research that focuses on educating providers about younger adult African American cancer survivors is critical, as is the development of community‐informed interventions focused on health literacy and the promotion of a healthy lifestyle to improve long‐term quality of life following completion of treatment.

## AUTHOR CONTRIBUTIONS

Conceptualization (MRT and AGS), formal analysis (MRT, JMK, and CML), funding acquisition (AGS and JLB), investigation (MRT, JMK, CML, JJR, TAH, FWKH, JLB, and AGS), methodology (MRT, JMK, and CML), project administration (JLB and AGS), writing ‐ original draft (MRT, JMK, and AGS), and writing ‐ review and editing (MRT, CML, JJR, TAH, FWKH, HT, JLB, JMK, and AGS).

## FUNDING INFORMATION

This work was supported by the National Cancer Institute of the National Institutes of Health (U01CA199240), the Epidemiology Research Core and the National Cancer Institute Center Grant (P30CA022453) to the Karmanos Cancer Institute at Wayne State University.

## CONFLICT OF INTEREST

The authors do not have any conflict of interest.

## ETHICS STATEMENT

The Karmanos Cancer Institute Protocol and Monitoring Review Board and the Wayne State University Institutional Review Board (#050417M1F) reviewed and approved this research with all participants in the Detroit ROCS study providing informed consent.

## Supporting information


Supplemental Table 1
Supplemental Table 2

## Data Availability

The data that support the findings of this study are available from the corresponding author upon reasonable request.
