# Supplementary material for: Evaluation of health behaviors and overall quality of life in younger adult African American cancer survivors
Source: Cancer Med. 2022 Jun 2;12(1):684–95. doi: 10.1002/cam4.4855 (PMC9844626; doi:10.1002/cam4.4855)
Supplement: Supplementary file 1 — Supplemental Table 1 Supplemental Table 2 [file CAM4-12-684-s001.docx]

Supplemental Materials to:

**Evaluation of Health Behaviors and Overall Quality of Life in Younger Adult African American Cancer Survivors**

Matthew R. Trendowski^1^, Jaclyn M. Kyko^1,2^, Christine M. Lusk^1,2^, Julie J. Ruterbusch^1,2^, Theresa A. Hastert^1,2^, Felicity W.K. Harper^1,2^, Hayley Thompson^1,2^, Jennifer L. Beebe-Dimmer^1,2^, and Ann G. Schwartz^1,2^

^1^Wayne State University School of Medicine, Department of Oncology, 4100 John R., Detroit, MI, USA; ^2^Karmanos Cancer Institute, 4100 John R., Detroit, MI, USA

**Supplemental Table 1: Evaluation of Sociodemographic, Behavioral, and Clinical Characteristics Associated with FACT-G or FACT-Cog in Univariate Analysis.**

|  | **β (95% CI)** | **p** |
| --- | --- | --- |
| **FACT-G** | | |
| Education | 3.17 (1.96, 4.38) | < 0.0001 |
| Marital Status |  |  |
| Married | Ref | Ref |
| Living with Partner | -9.42 (17.84, -1.007) | 0.03 |
| Widowed | -19.96 (-32.85,-7.06) | 0.003 |
| Divorced | -9.33 (-15.22, -3.43) | 0.002 |
| Separated | -10.76 (-21.08,-0.44) | 0.04 |
| Never Married | -9.90 (-14.43, -5.37) | < 0.0001 |
| Income Level (one-category increase) | 6.28 (4.75, 7.80) | < 0.0001 |
| Insurance Status |  |  |
| Medicare | Ref | Ref |
| Medicaid | -0.54 (-7.68, 6.60) | 0.88 |
| Private | 13.43 (6.31, 20.55) | 0.0002 |
| Involvement in Religious Activities | 2.70 (1.27, 4.12) | 0.0002 |
| Health Literacy Confidence (one-point increase) | 7.52 (5.75, 9.30) | < 0.0001 |
| Engaged in ≥150 Minutes of Physical Activity per Week |  |  |
| None | Ref | Ref |
| <150 min | 4.05 (-0.57, 8.68) | 0.09 |
| ≥150 min | 8.03 (3.53, 12.55) | 0.0005 |
| Consumed ≥5 Fruits and Vegetables per Day | 1.51 (-2.66, 6.57) | 0.48 |
| Smoking Status |  |  |
| Current | Ref | Ref |
| Former | 6.76 (-0.03, 13.55) | 0.05 |
| Never | 11.01 (5.33, 16.70) | 0.0001 |
| Alcohol Consumption (yes vs no) | 3.23 (-0.59, 7.04) | 0.10 |
| BMI at Enrollment | -0.15 (-0.35, 0.05) | 0.15 |
| Total Comorbidity Count | -4.45 (-5.89, -3.01) | < 0.0001 |
| Ever Perceived Discrimination | -5.77 (-9.54, -1.99) | 0.003 |
| **FACT-Cog** | | |
| Education | 1.16 (-0.78, 3.11) | 0.24 |
| Marital Status |  |  |
| Married | Ref | Ref |
| Living with Partner | -1.32 (-9.81, 7.16) | 0.76 |
| Widowed | -3.13 (17.27, 11.00) | 0.66 |
| Divorced | -8.87 (14.94, -2.79) | 0.004 |
| Separated | -0.24 (-12.62, 12.12) | 0.96 |
| Never Married | -2.02 (-6.55, 2.51) | 0.38 |
| Income Level (one-category increase) | 2.09 (0.52, 3.68) | 0.009 |
| Insurance Status |  |  |
| Medicare | Ref | Ref |
| Medicaid | 2.33 (-5.27, 9.93) | 0.54 |
| Private | 4.37 (-3.21, 11.95) | 0.26 |
| Involvement in Religious Activities | 1.46 (0.02, 2.91) | 0.05 |
| Health Literacy Confidence (one-point increase) | 5.52 (3.71, 7.33) | < 0.0001 |
| Engaged in≥150 Minutes of Physical Activity per Week |  |  |
| None | Ref | Ref |
| <150 min | 0.21 (-4.40, 4.83) | 0.93 |
| ≥150 min | 0.13 (-4.49, 4.74) | 0.96 |
| Consumed ≥5 Fruits and Vegetables per Day | 2.00 (-2.18, 6.17) | 0.35 |
| Smoking Status |  |  |
| Current | Ref | Ref |
| Former | 4.49 (-2.57, 11.56) | 0.21 |
| Never | 6.29 (0.72, 11.86) | 0.03 |
| Alcohol Consumption (yes vs no) | -3.08 (-6.86, 0.69) | 0.11 |
| BMI at Enrollment | 0.03 (-0.15, 0.22) | 0.72 |
| Total Comorbidity Count | -2.74 (-4.11, -1.37) | 0.0001 |
| Ever Perceived Discrimination | -3.29 (-7.05, 0.48) | 0.09 |

**Supplemental Table 2. Evaluation of Sociodemographic and Clinical Characteristics Associated with PROMIS^®^ Anxiety or Depression in Univariate Analysis.**

|  | **OR (95% CI)** | **p** |
| --- | --- | --- |
| **PROMIS^®^ Anxiety** | | |
| Education | 0.86 (0.70, 1.06) | 0.15 |
| Marital Status |  |  |
| Married | Ref | Ref |
| Living with Partner | 2.00 (0.75, 5.00) | 0.15 |
| Widowed | 3.39 (0.93, 11.57) | 0.05 |
| Divorced | 1.93 (0.99, 3.78) | 0.05 |
| Separated | 2.76 (0.94, 7.67) | 0.05 |
| Never Married | 2.08 (1.23, 3.61) | 0.007 |
| Income Level (one-category increase) | 0.78 (0.37, 0.97) | 0.006 |
| Insurance Status |  |  |
| Medicare | Ref | Ref |
| Medicaid | 0.50 (0.24, 1.06) | 0.07 |
| Private | 0.38 (0.18, 0.81) | 0.01 |
| Involvement in Religious Activities | 0.88 (0.76, 1.04) | 0.13 |
| Health Literacy Confidence (one-point increase) | 0.66 (0.55, 0.81) | < 0.0001 |
| Engaged in ≥150 Minutes of Physical Activity per Week |  |  |
| None | Ref | Ref |
| <150 min | 1.003 (0.62, 1.62) | 0.99 |
| ≥150 min | 0.61 (0.36, 1.006) | 0.06 |
| Consumed ≥5 Fruits and Vegetables per Day | 1.13 (0.74, 1.79) | 0.56 |
| Smoking Status |  |  |
| Current | Ref | Ref |
| Former | 0.75 (0.38, 1.49) | 0.41 |
| Never | 0.49 (0.28, 0.89) | 0.02 |
| Alcohol Consumption (yes vs no) | 1.12 (0.75, 1.68) | 0.57 |
| BMI at Enrollment | 1.01 (0.99, 1.03) | 0.24 |
| Total Comorbidity Count | 1.16 (0.99, 1.35) | 0.07 |
| Ever Perceived Discrimination | 1.56 (0.37, 0.97) | 0.03 |
| **PROMIS^®^ Depression** | | |
| Education | 0.79 (0.63, 0.98) | 0.03 |
| Marital Status |  |  |
| Married | Ref | Ref |
| Living with Partner | 3.69 (1.32, 9.96) | 0.01 |
| Widowed | 4.62 (1.11, 16.94) | 0.02 |
| Divorced | 3.02 (1.41, 6.68) | 0.005 |
| Separated | 3.55 (1.01, 11.20) | 0.04 |
| Never Married | 3.19 (1.72, 6.33) | 0.0005 |
| Income Level (one-category increase) | 0.66 (0.42, 1.15) | < 0.0001 |
| Insurance Status |  |  |
| Medicare | Ref | Ref |
| Medicaid | 0.49 (0.23, 1.04) | 0.06 |
| Private | 0.28 (0.13, 0.61) | 0.001 |
| Involvement in Religious Activities | 0.86 (0.73, 1.01) | 0.07 |
| Health Literacy Confidence (one-point increase) | 0.59 (0.48, 0.73) | < 0.0001 |
| Engaged in ≥150 Minutes of Physical Activity per Week |  |  |
| None | Ref | Ref |
| <150 min | 0.75 (0.44, 1.27) | 0.29 |
| ≥150 min | 0.63 (0.37, 1.08) | 0.10 |
| Consumed ≥5 Fruits and Vegetables per Day | 0.88 (0.56, 1.41) | 0.59 |
| Smoking Status | 0.72 (0.54, 0.97) | 0.03 |
| Current | Ref | Ref |
| Former | 0.88 (0.43, 1.83) | 0.73 |
| Never | 0.55 (0.30, 1.04) | 0.06 |
| Alcohol Consumption (yes vs no) | 1.09 (0.71, 1.67) | 0.71 |
| BMI at Enrollment | 1.02 (1.00, 1.05) | 0.03 |
| Total Comorbidity Count | 1.39 (1.18, 1.63) | < 0.0001 |
| Ever Perceived Discrimination | 1.32 (0.42, 1.15) | 0.22 |
